# Seroprevalence and Antibody Magnitude of *Brucella canis* in Shelter Dogs: A Four-Year Study in Southern Italy

**DOI:** 10.3390/vetsci13040315

**Published:** 2026-03-25

**Authors:** Valentina Iovane, Elvira Improda, Antonella Rossi, Giuseppe Iovane, Ugo Pagnini, Nebyou Moje Hawas, Roberto Ciarcia, Serena Montagnaro

**Affiliations:** 1 Department of Agricultural Science, University of Naples Federico II, 80055 Portici, Italy; valentina.iovane@unina.it; 2 Department of Veterinary Medicine and Animal Productions, University of Naples Federico II, 80137 Naples, Italy; elviraviolaimproda@gmail.com (E.I.); upagnini@unina.it (U.P.); 3 Experimental Zooprofilactic Institute of Southern Italy, 80055 Portici, Italy; iovane@unina.it; 4 Department of Biomedical Science, College of Veterinary Medicine and Agriculture, Addis Ababa University, Bishoftu (Debre Zeit), Ethiopia; nebyou.moje@aau.edu.et

**Keywords:** *Brucella canis*, seroprevalence, IFAT, province, Southern Italy, year

## Abstract

**Background**: *Brucella canis* is an emerging zoonotic pathogen responsible for canine reproductive disorders and public health concerns. This study assessed the seroprevalence of *B. canis* in dogs from Campania, Southern Italy (2022–2025). **Methods**: Serum samples (n = 400) were retrospectively screened using an indirect immunofluorescence assay (IFAT), performed according to the manufacturer’s instructions. Screening was conducted at a 1:40 cut-off, followed by serial dilutions to determine endpoint titres. Statistical analysis included chi-square tests for univariable screening, followed by nominal logistic regression models to evaluate the association between IFAT positivity and predictive factors (year, province, and sex of dogs). Additionally, a general linear model (GLM) was applied to the seropositive subset (n = 69) to analyse the magnitude of the antibody response, expressed as geometric mean titres (GMTs). **Results:** The overall seroprevalence was 17.3% (95% CI: 13.6–21.0%). Dog’s sex, year of sampling, and province were not significant independent predictors of infection (*p* > 0.05), but GLM analysis showed that sampling year (*p* = 0.0024) and province (*p* = 0.0490) significantly influenced antibody intensity. A significant temporal increase in antibody intensity was observed towards 2025 (*p* = 0.037), suggesting an intensification of infection pressure. **Conclusions:** Our results confirm that *Brucella canis* is an endemic pathogen in the shelter dog population of southern Italy. The high seroprevalence and significant increase in antibody magnitude (GMT) over the study period indicate rising infection pressure, highlighting the urgent need for mandatory screening and a coordinated One Health surveillance strategy to manage zoonotic risk effectively.

## Simple Summary

Canine brucellosis, caused by the bacterium *Brucella canis*, is a disease that affects the reproductive system of dogs and can be transmitted to humans, posing a threat to public health. Despite its significance, this infection is often overlooked in Italy, and there are no national mandatory monitoring plans in shelters. This study examined the prevalence of the pathogen in 400 dogs housed in shelters in the Campania region (Southern Italy) between 2022 and 2025. The results show that over 17% of the animals had been exposed to the bacterium, a much higher percentage than observed in other areas of Italy. Although the number of positive dogs remained stable over time, the strength of the immune response increased significantly towards 2025, suggesting increasingly active circulation of the pathogen. These findings are of great value to society, as they highlight the need for mandatory screening of dogs entering shelters or being adopted to protect the health of the animals, their owners, and shelter staff.

## 1. Introduction

Brucellosis is a global bacterial zoonosis caused by members of the genus *Brucella*, which can infect humans, wildlife, companion animals, and livestock [1,2,3]. This disease represents a persistent public health threat, exacerbated by the absence of a protective vaccine for humans and the inherent limitations of current animal vaccination strategies [4,5,6,7]. Although brucellosis caused by *B. abortus* and *B. melitensis* has historically been associated with cattle and small ruminants, respectively, emerging evidence indicates that domestic carnivores, such as dogs and cats, may play a central role in the spread and re-emergence of other *Brucella* species [8].

One specific species of *Brucella*, *Brucella canis*, is a Gram-negative, rough-type lipopolysaccharide (LPS) coccobacillus and a significant pathogen in both dogs and humans [9]. Although domestic dogs are the primary hosts, serological studies have identified positive cases in wild canids, such as foxes and jackals [10]. Conversely, experimental studies on cats, pigs, and ruminants have shown that these species possess a high degree of resistance to *B. canis* infection, failing to develop significant antibody titres or persistent infections [1].

From a clinical and economic perspective, *B. canis* is a leading cause of canine infertility [11]. Horizontal transmission is frequently facilitated by environmental contamination within animal shelters, resulting in substantial reproductive losses [12,13]. Clinical manifestations in dogs include orchitis, epididymitis, endocarditis, uveitis, and discospondylitis [8]. In humans, the clinical progression of *B. canis* is virtually identical to that observed in *B. abortus* infections. The disease typically manifests with an insidious onset and is characterised by recurrent relapses, although it rarely transitions into a chronic state [14]. The zoonotic human infection typically presents as fluctuating fever, lymphadenopathy, and splenomegaly, though untreated cases can progress to severe systemic complications involving the central nervous system or endocardium [8]. The diagnostic challenge of neurobrucellosis was recently highlighted in Japan, where diagnosis was confirmed by cerebrospinal fluid analysis in a patient with unexplained neurological symptoms [15]. The risk of domestic transmission was emphasised by a notable case in the United States, where a three-year-old child was infected by a terrier puppy [1].

The current landscape of globalisation, marked by an exponential increase in the international movement of pets from commercial breeding, overseas adoption campaigns, and humanitarian crises, has accelerated the transboundary spread of *B. canis* [8,16,17,18,19]. In particular, the transboundary movement of dogs from endemic regions poses a substantial risk for the introduction and dissemination of *B. canis*. Consequently, several countries, most notably the United Kingdom, have emphasised the necessity of implementing more stringent import controls and risk-based screening. Such interventions are critical to identify infected carriers and prevent the pathogen from becoming established in previously non-endemic territories [19,20]. Italy is not exempt from these dynamics, as the increasing influx of dogs from potentially endemic areas of Eastern Europe and the Balkan Peninsula necessitates a more thorough assessment of the national epidemiological situation. In Italy, while brucellosis caused by *B. abortus* and *B. melitensis* is subject to rigorous national eradication programmes in livestock, *B. canis* remains a neglected pathogen under the current legislative framework. Although the Italian Veterinary Police Regulation [21] requires the reporting of zoonotic diseases, there is no specific national monitoring plan or mandatory screening protocol for canine brucellosis in kennels. Italian legislation does not currently require mandatory screening for *B. canis* in all kennels or for privately owned dogs, as it is not considered an infectious disease subject to a national eradication plan, unlike other brucellae. However, specific controls are mandatory in the event of a confirmed outbreak [22].

Recent reports of *B. canis* in Europe rely mainly on sporadic observations of clinical manifestations [12,23,24]. The lack of systematic investigations or large-scale cross-sectional studies, combined with the absence of harmonised surveillance protocols, prevents accurate mapping of the pathogen’s endemicity within the European continent. This gap limits the effectiveness of evidence-based public health interventions at the regional level.

To address these limitations, the present study sought to investigate the spatiotemporal dynamics and seroprevalence of *B. canis* within a specific Mediterranean context. Specifically, this work aimed to evaluate the circulation of the pathogen in dogs in the Campania region (Southern Italy) through a temporal survey analysis spanning from 2022 to 2025.

## 2. Materials and Methods

### 2.1. Ethical Statement

This study was conducted in accordance with national and international animal welfare guidelines. Ethical approval was not required, as the research used only residual serum aliquots. The serum samples used in this study were obtained from stray dogs housed in municipal shelters during institutional health screenings. These protocols, including comprehensive blood profiling and infectious disease monitoring, were conducted by the official Veterinary Services (Local Health Units—ASL) as part of mandatory regional surveillance programmes. This large-scale screening took place during routine veterinary examinations for annual vaccinations or periodic inspections, in accordance with Regional Law no. 16, 24 November 2001 [25]. No animals underwent additional sampling or procedures for this study. All diagnostic activities formed part of institutional surveillance programmes, and the use of leftover materials for research complies with national legislation regarding the secondary use of clinical samples.

### 2.2. Study Area and Sample Size

The study was conducted in the Campania region of Southern Italy (41°00′00″ N, 14°30′00″ E), which covers an area of 13,595 km^2^ and has a 350 km coastline along the Tyrrhenian Sea. The regional climate is Mediterranean in coastal areas and continental in the mountainous hinterland [26]. The target population comprised dogs housed in 49 shelters across the five provinces of the region (Avellino, Benevento, Caserta, Naples, and Salerno). Under current local health protocols, serological screening for *B. canis* is not mandatory at shelter admission. Consequently, animals were sampled during their stay, representing a heterogeneous group of recent arrivals and long-term residents. This allowed for an assessment of the overall infectious pressure within the shelter system, where the lack of entry testing may contribute to the persistence of the pathogen.

The sample size was calculated using the formula for a theoretically infinite population [27]. In the absence of prior epidemiological data specific to the Campania region and considering that existing Italian reports (limited to the Northeast) indicated a very low prevalence (0.0–1.95%) [19,28], a conservative expected prevalence of 50% was assumed to ensure maximum statistical power. With a 95% confidence interval and an absolute precision of 5%, a minimum of 385 dogs was required; a total of 400 dogs was ultimately sampled.

From January 2022 to December 2025, blood samples were collected during routine sanitary screenings conducted by the official government veterinary services. A total of 400 serum samples were obtained from dogs residing in 49 authorised shelters distributed as follows: Caserta (n = 13), Avellino (n = 11), Naples (n = 10), Salerno (n = 9), and Benevento (n = 6). This distribution reflects the institutional network of long-term shelters under the surveillance of the respective Local Health Units (ASL). The sampling strategy involved simple random sampling of at least 100 canine serum samples for each year of the study period (2022 to 2025). To minimise selection bias and ensure that results were not disproportionately influenced by individual facility management, samples were randomly selected from the available institutional pool each year. For each dog included in the study, the geographical area and sex were systematically recorded.

### 2.3. Sample Preparation and Serological Analysis

Individual blood samples were collected from each dog during clinical examinations for routine health checks, annual immunisation protocols, or official inspections. Residual serum aliquots were subsequently stored at −80 °C at the Unit of Infectious Disease, Department of Veterinary Medicine and Animal Production, University of Naples “Federico II”. To ensure protein stability and maintain diagnostic integrity, only samples subjected to no more than three freeze–thaw cycles were used in this study [29].

All serum samples were screened for specific IgG antibodies against *B. canis* using a commercial indirect immunofluorescence antibody test (IFAT) (MegaFLUO^®^ BRUCELLA canis, MEGACOR Diagnostik GmbH, Hörbranz, Austria). The assay performance, as reported by Perletta et al. [30], is characterised by a sensitivity of 98.4%, a specificity of 99.3%, and an accuracy of 99.0%. Given the high performance of the assay, the apparent prevalence was considered a reliable proxy for the true prevalence in this population. The assay was performed according to the manufacturer’s instructions. A two-step diagnostic protocol was implemented: first, all sera were diluted in phosphate-buffered saline (PBS, pH 7.2–7.4) and screened at a 1:40 dilution, which served as the designated cut-off for positivity.

Samples that tested positive in this preliminary screening were retested and subjected to a complete two-fold serial dilution series (1:40, 1:80, 1:160, and 1:320) in PBS to determine the final endpoint titre, defined as the highest dilution still exhibiting visible fluorescence. For each test run, twenty microlitres (20 µL) of the diluted sample, together with the provided positive and negative controls, were applied to the antigen-coated wells. After a 30 min incubation at 37 °C in a humid chamber, the slides were washed twice with PBS and rinsed with distilled water. FITC anti-dog IgG conjugate was then applied and incubated for a further 30 min at 37 °C in the dark.

Microscopic examination was performed independently by three operators using a fluorescence microscope (Leica DM 2500, Wetzlar, Germany). Fluorescence patterns were visualised at the appropriate magnification to match the kit’s requirements (400× equivalent), using the green fluorescence channel (excitation/emission: 480/517 nm) compatible with the FITC conjugate. A sample was confirmed as positive when the coccobacilli displayed a distinct yellow-green fluorescence pattern identical to the positive control at a dilution of ≥1:40. Any reaction patterns differing from the positive control were interpreted as nonspecific and recorded as negative.

### 2.4. Statistical Analysis

Statistical analyses were conducted to evaluate the association between the categorical predictors (year, province, and dog’s sex) and *B. canis* seropositivity. The shelter environment was not included as a comparative risk factor because the study focused exclusively on dogs already housed in these facilities. The lack of a non-shelter control group, such as owned dogs, prevented formal statistical measurement of the “shelter” variable. Univariable analysis (chi-square) was performed first, followed by a multivariable logistic regression model to estimate adjusted odds ratios (aORs) and identify independent predictors of seropositivity risk. To assess whether epidemiological factors influenced the magnitude of the immune response, a sub-analysis was conducted on the cohort of seropositive dogs (N = 69) for which endpoint titres were available. The antibody titres were converted into a continuous response variable, defined as log2-transformed titres, by applying a log2 transformation to the reciprocal of the endpoint dilution (Y = log2[titre]). This approach was used to linearise the progression of the serological data and satisfy the normality assumptions required for parametric testing. The model results were then back-transformed to the original scale and presented as adjusted geometric mean titres (GMTs) with their 95% confidence intervals. A general linear model (GLM) was employed, specifying a normal distribution and an identity link function, with log2-transformed titres as the dependent variable. The model included year, province, and dog’s sex as fixed effects. For factors demonstrating a significant main effect in the global test, least squares means (LS means) were compared using custom contrast tests. Specifically, comparisons were made between the initial sampling year (2022) and the reference sampling year (2025), as well as between provinces with extreme estimates relative to the reference province, Salerno. Statistical significance was set at *p* < 0.05.

Statistical analyses were performed using JMP Student Edition software, version 18.2.2 (SAS Institute Inc., Cary, NC, USA). Geographical distribution maps were created using the web-based visualisation software Datawrapper (Datawrapper GmbH, Berlin, Germany; https://www.datawrapper.de, accessed on 23 January 2026).

## 3. Results

### 3.1. Seroprevalence and Risk Factor Analysis

The 400 serum samples were collected over a four-year period (2022–2025) from 49 authorised shelters, comprising 222 males (55.5%) and 178 females (44.5%). For geographical distribution, the largest proportion of samples came from the province of Caserta (28.3%, n = 113, collected from 13 shelters), followed by Naples (24.3%, n = 97, from 10 shelters), Salerno (21.0%, n = 84, from 9 shelters), Avellino (17.0%, n = 68, from 11 shelters), and Benevento (9.5%, n = 38, from 6 shelters).

**Figure 1:**
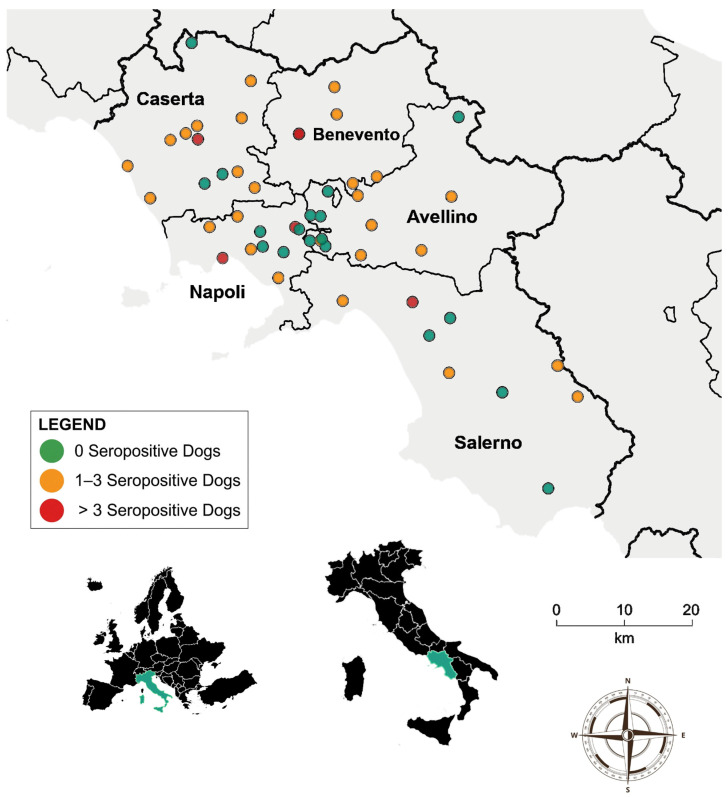
Spatiotemporal distribution of *Brucella canis* seropositive dogs in the Campania region, Italy (2022–2025). The map shows the distribution of positive cases across the five provinces in the study area (Avellino, Benevento, Caserta, Naples, and Salerno). Sampling locations are indicated by circles, with different colours representing specific count intervals of detected seropositive dogs: green for zero, orange for 1–3, and red for more than three cases. The inset maps show the region’s geographical position within Italy and Europe. A scale bar and compass rose are included for reference.

The overall seroprevalence of *B. canis* in the studied population was 17.3% (69/400) (Figure 1, Table 1). Univariable analysis showed that neither year (*p* = 0.3634), dog’s sex (*p* = 0.9565), nor province (*p* = 0.498) was significantly associated with *B. canis* seropositivity (Table 1). For the dog’s sex, the crude prevalence rates were 16.9% for females and 17.6% for males (Table 1). Regarding geographical distribution, although Benevento showed a higher crude risk compared to Salerno (OR = 2.37; 95% CI: 0.91–6.21), no statistically significant differences were found between provinces (Table 1). The geographical distribution of positive cases was widespread, with at least one positive dog detected in each province, although the magnitude of infection varied between facilities (Figure 1). Furthermore, the multivariable logistic regression model confirmed the absence of statistically significant independent predictors for *B. canis* seropositivity (Table 2). However, two relevant trends were observed. Firstly, the province of Benevento exhibited a higher adjusted risk (aOR = 2.49; 95% CI: 0.85–7.29; *p* = 0.0961) compared to that of Salerno. The risk of *B. canis* seropositivity in 2022 did not differ significantly from the 2025 reference year, despite a lower adjusted odds ratio (aOR = 0.45; 95% CI: 0.20–1.01; *p* = 0.0533).

### 3.2. Magnitude of Antibody Response (IFAT Titres)

**Figure 2:**
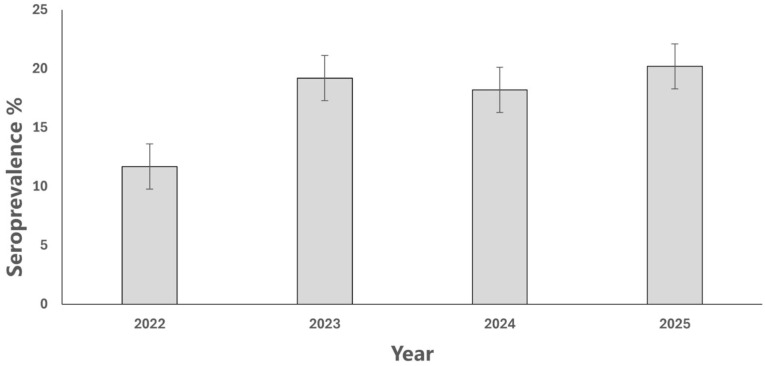
Annual seroprevalence of *B. canis* in Campania (2022–2025). Bars represent the percentage of seropositive dogs with 95% confidence intervals. No significant temporal trend in seroprevalence was observed (*p* = 0.3634).

**Figure 3:**
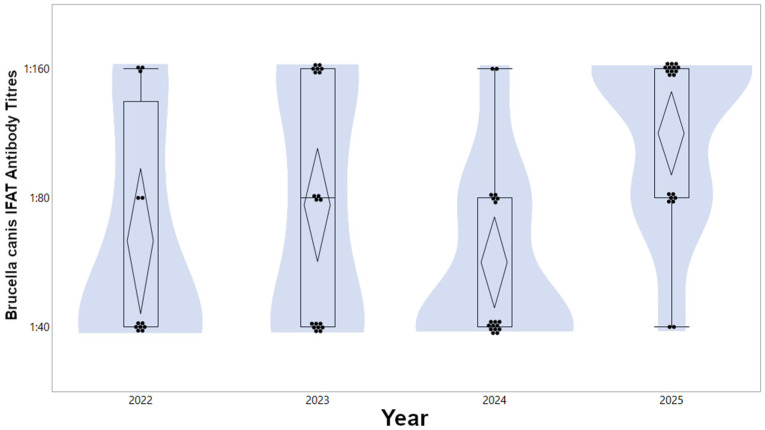
Distribution of *B. canis* IFAT antibody titres by sampling year. Individual data points are displayed with a jitter effect; violin plots show the density distribution of titres. The horizontal lines within the central diamonds indicate the adjusted geometric mean titres (GMTs), while the vertical extent of the diamonds represents the 95% confidence intervals. The y-axis is shown on a log2-transformed scale, with labels reflecting the original twofold serial dilutions (1:40, 1:80, 1:160).

Analysis of the antibody titre intensity in seropositive dogs (n = 69; 17.3%) revealed that, while epidemiological factors did not significantly predict the presence of infection status, they significantly influenced the magnitude of the immune response (Figure 2). The general linear model (GLM) performed on log2-transformed titres showed a significant main effect for both year (*p* = 0.0024) and province (*p* = 0.0490) (Table 3, Figure 3). Post hoc contrast tests for the temporal effect revealed a significant difference in *B. canis* seropositive results between those sampled in 2022 and the 2025 reference year (chi-square = 4.33; *p* = 0.037). Specifically, the adjusted geometric mean titre (GMT) in 2022 was significantly lower than in 2025 (mean log2 difference: −0.317) (Figure 3). Regarding geographical distribution, although the global test was significant, pairwise contrasts between the provinces with extreme estimates did not reach statistical significance (*p* > 0.05). The dog’s sex did not influence the antibody magnitude in seropositive subjects (*p* = 0.6588).

## 4. Discussion

This study reveals a substantial seroprevalence of *B. canis* (17.3%) in the shelter dog population of Campania, comprising animals originally rescued as free-roaming or stray dogs. This figure is significantly higher than those recently reported in other European contexts, such as the United Kingdom and Germany, where the pathogen is often associated with imported animals [8,18,19].

The estimated mean prevalence of *B. canis* in Europe is 4.7% [31], although significant geographical variation exists depending on the population studied. Notably, the seroprevalence observed in the Campania region (17.3%) is consistent with findings from shelter dog populations in Egypt (15%), meta-analyses conducted in Belgium (12.2%), and retrospective diagnostic data from Turkey (12.7%) [31,32,33]. Higher rates, approaching 30%, have been reported in specific cohorts in the Netherlands and Switzerland, while significantly lower values have been recorded in France (2.7%) and Germany (5.4%) based on meta-analyses and retrospective diagnostic data [31,32]. A marked epidemiological disparity is evident in Italy: our findings in southern Italy significantly exceed the lowest prevalence rates (0.0–1.95%) recently documented in northeastern and central Italy, where studies have focused on specific cohorts such as shelter dogs, blood donors, and dogs belonging to Ukrainian refugees [19,28]. Furthermore, these findings surpass the 7.9–9.25% range reported in larger meta-analyses [31,32], identifying the study region as a high-pressure endemic area. The use of a conservative 50% expected prevalence during the study design phase proved appropriate, as the detected seroprevalence (17.3%) significantly exceeded the sporadic figures previously reported in Northern Italy. This underscores the importance of regional-scale epidemiological studies, as low-prevalence estimates from geographically distant areas may substantially underestimate the true pathogen burden in endemic Mediterranean contexts. Such disparities may result from regional differences in stray dog management, population density within shelters, or specific environmental factors that favour the persistence of the pathogen in Southern Italy compared to Northern regions. It is important to note that the subjects in our cohort entered the facilities as unidentified strays and were microchipped only upon admission; thus, while they are categorised as shelter dogs for the purposes of this study, their serological status reflects both environmental exposure in the territory and potential transmission during their residence in the facilities. However, these findings require cautious interpretation, as the diagnosis of *B. canis* remains problematic due to the inherent limitations of current diagnostic protocols [34]. Accurate identification typically requires a multimodal approach, integrating various serological assays with molecular techniques and gold-standard bacterial isolation [30]. Although IFAT is a widely accepted screening tool due to its high sensitivity, the potential for cross-reactivity with other bacteria (including other rough Brucella species such as *B. ovis* and, to a lesser extent, bacteria such as *Yersinia enterocolitica* and *Escherichia coli*) has been recognised as an intrinsic limitation of the serological protocol [30]. Additionally, the intrinsic biological and pathophysiological characteristics of the bacterium, specifically its facultative intracellular lifestyle and intermittent shedding, hinder diagnostic sensitivity [35]. These factors, along with often nonspecific clinical manifestations, contribute to the frequent underdiagnosis of the infection in routine veterinary surveillance, resulting in few case reports [28]. However, comparisons across different studies warrant cautious interpretation, as the present work utilised a commercial IFAT for *B. canis* antibody detection, whereas the available literature relies on a wide range of serological and molecular assays [36]. These methodological discrepancies are critical, as different diagnostic tests exhibit varying levels of sensitivity and specificity. Furthermore, the absence of a universally recognised gold standard method for canine brucellosis screening complicates direct comparison of prevalence rates across different geographical areas and study populations [37].

Furthermore, although the high-density nature of shelters suggests they may act as risk factors for *B. canis* transmission, our cross-sectional design does not allow formal differentiation between infections acquired as stray dogs in the territory and those potentially occurring after shelter entry. Identifying the shelter as an independent risk factor would have required a prospective longitudinal approach, including mandatory testing with a gold standard assay upon admission to ensure a negative baseline for all subjects. Without such baseline data, any conclusion regarding the direction of transmission remains speculative and was considered beyond the scope of the present study. The exact site of infection, whether the territory or the shelter environment, therefore, remains unknown. Additionally, specific facility-level management indicators, such as overcrowding and turnover rates, were unavailable for analysis due to jurisdictional and data access constraints, as this information is strictly limited to internal health authority administration. Nevertheless, the environment remains a critical point for epidemiological surveillance.

In our cohort, the lack of a significant association between seropositivity and predictive factors (year, province, and dog’s sex) in the multivariable model suggests that *B. canis* has established an endemic status within the regional territory. Although the province of Benevento showed a higher adjusted risk (aOR = 2.49) than Salerno, the lack of statistical significance in the multivariable model (*p* = 0.0961) suggests that the pathogen’s distribution is now homogeneous across the region. However, the high odds ratio observed in Benevento, despite the small sample size (n = 38), warrants attention, as it may indicate specific epidemiological hotspots or different management dynamics of stray populations in the area. Nevertheless, the widespread distribution of *B. canis* seropositivity across all five provinces indicates that transmission cycles are active and not confined to specific geographical or demographic clusters, suggesting a consolidated environmental and behavioural reservoir within the stray dog population.

A key finding of this research is the distinction between the prevalence of exposure and the magnitude of the immune response. While the probability of being seropositive remained statistically stable over the four-year period, the GLM identified a significant temporal increase in antibody intensity. In Figure 3, the adjusted GMT results and their 95% confidence intervals are shown as confidence diamonds. The rising trend in adjusted GMT towards 2025 suggests an intensification of infectious pressure. This hypothesis is supported by the highly variable antibody kinetics of *B. canis*. Although antibodies may persist for months after infection resolves, their levels physiologically decline as bacteraemia subsides, and the infection stabilises into a chronic or localised state (e.g., prostate and epididymides) [38]. Consequently, while low or moderate titres often reflect remote exposure or late-stage chronic infection, the high titres observed in the final years of the study more likely indicate the circulation of recent infections or active bacteraemic phases within the territory [39]. This phenomenon could be attributed to more frequent re-exposures resulting from the chronicity of the infection in residents or to the possible circulation of strains with a greater capacity for immunogenic stimulation. The high seroprevalence (17.3%) detected across all provinces identifies the region as a high-pressure endemic area, significantly exceeding the rates documented in Northern and Central Italy [19,28]. Given that infected dogs can remain asymptomatic while intermittently shedding the bacterium [35], rigorous pre-movement testing is essential to prevent the accidental introduction of *B. canis* into non-endemic households or geographical areas.

From a One Health perspective, these findings represent a significant public health concern in the area. The proximity of these rescued dogs, originally strays and now kennel residents, to shelter personnel, combined with the lack of mandatory screening in Italian kennels, creates a high-risk environment for spillover events. Therefore, our study indicates that a systematic surveillance programme integrating serological screening and molecular confirmation is urgently required to mitigate the risk to both the canine population and human caregivers.

## 5. Conclusions

The findings of this four-year surveillance study confirm that Brucella canis is endemic in the shelter dog population in Campania, which is the main result of the study. Although the overall seroprevalence (17.3%) did not show significant annual fluctuations in the multivariable model, the most notable result is the significant and progressive increase in antibody magnitude (GMT) recorded between 2022 and 2025. This trend indicates increasing infection pressure and active pathogen circulation within multi-dog environments, where overcrowding and high turnover facilitate transmission. Given the zoonotic potential of *B. canis* and the high risk of accidental introduction into households through adoptions, these results have immediate implications for public health and veterinary policy. Mandatory screening programmes, requiring systematic serological testing for all dogs upon entry into the shelter system, particularly in high-prevalence regions, should be implemented. Furthermore, there is an urgent need for standardised national guidelines to regulate the management of seropositive individuals, including rigorous isolation protocols and compulsory notification of cases to the relevant health authorities.

Finally, consistent with a One Health framework, strengthening collaboration between veterinary services and public health professionals is essential. Such cooperation is indispensable for effective surveillance of potential spillover events involving both shelter personnel and adoptive families, thereby ensuring comprehensive protection of human and animal health. In conclusion, while prevalence remains stable, the rising intensity of the humoral response signals a growing biological threat. Shifting the focus from detection to analysis of infection magnitude provides a more comprehensive tool for assessing the epidemiological risk of canine brucellosis in endemic areas.

## Figures and Tables

**Table 1:** Descriptive seroprevalence of *B. canis* infection in the study population (n = 400), stratified by sampling year, province, and sex of the dogs. Grey shading highlights category headers.

Factor	n	Positive	%	SE % *	95%CI ^#^	χ2	*p*-Value
Total	400	69	17.3	8.7	13.6–20.9		
Year							
2022	103	12	11.7	6.2	5.5–17.9	3.189	0.3634
2023	99	19	19.2	7.8	11.4–26.9		
2024	99	18	18.2	7.6	10.6–25.8		
2025	99	20	20.2	7.9	12.3–28.1		
Province							
Avellino	68	11	16.2	8.8	7.4–24.9		
Benevento	38	10	26.3	14.0	12.3–40.3		
Caserta	113	19	16.8	6.9	9.9–23.7	3.390	0.498
Naples	97	18	18.6	7.7	10.8–26.3		
Salerno	84	11	13.1	7.2	5.9–20.3		
Sex							
Male	222	39	17.6	5.0	12.6–22.6	0.003	0.9565
Female	178	30	16.9	7.2	11.4–22.4		

* Standard error; ^#^ 95% confidence intervals.

**Table 2:** Multivariable analysis with adjusted odds ratios (aOR) for predictors of *B. canis* seropositivity in Campania (2022–2025).

Predictor	Category	Log-Odds Estimate	aOR *	95% CI ^#^	*p*-Value
Province	Salerno	Ref.	Ref.	Ref.	Ref.
	Benevento	0.9120	2.49	0.85–7.29	0.0961
	Avellino	0.4678	1.60	0.62–4.08	0.3290
	Naples	0.5392	1.72	0.75–3.94	0.2034
	Caserta	0.2679	1.31	0.56–3.07	0.5383
Year	2025	Ref.	Ref.	Ref.	Ref.
	2022	−0.8026	0.45	0.20–1.01	0.0533
	2023	−0.2595	0.77	0.34–1.73	0.5277
	2024	−0.2251	0.80	0.38–2.13	0.5562
Gender	Male (M)	Ref.	Ref.	Ref.	Ref.
	Female (F)	−0.1140	0.89	0.52–1.52	0.6758

* Adjusted odds ratio; ^#^ 95% confidence intervals.

**Table 3:** General linear model (GLM) results for antibody magnitude (log2-transformed titres) in seropositive dogs (n = 69).

Factor	χ2	DF ^¥^	*p*-Value	Significance ^$^
Year	14.45	3	0.0024 ***	Highly Significant
Province	9.54	4	0.0490 *	Significant
Dog’s sex	0.19	1	0.6588	Not Significant

^¥^ Degrees of freedom; ^$^ significant at *p* < 0.05; *** highly significant; * significant.

## Data Availability

The original contributions presented in this study are included in the article. Further inquiries can be directed to the corresponding authors.

## Abbreviations

The following abbreviations are used in this manuscript:

PBS	Phosphate Buffered Saline
IFAT	Indirect Immunofluorescence Antibody Test
FITC	Fluorescein Isothiocyanate
SE	Standard Error
CI	Confidence Interval
DF	Degrees of Freedom
OR	Odds Ratio
aOR	Adjusted Odds Ratio
GLM	General Linear Model
GMT	Geometric Mean Titre
